# Posttransplant Lymphoproliferative Disorder in a Patient with Worsening Ascites after Liver Transplantation

**DOI:** 10.1155/2017/7247438

**Published:** 2017-09-11

**Authors:** Harsh D. Patel, Moises I. Nevah Rubin

**Affiliations:** ^1^McGovern Medical School, Department of Internal Medicine, The University of Texas Health Science Center at Houston, 6431 Fannin St., Suite MSB 1.134, Houston, TX 77030, USA; ^2^McGovern Medical School, Department of Gastroenterology, Transplant and Advanced Hepatology, The University of Texas Health Science Center at Houston, 6431 Fannin St., Suite MSB 1.134, Houston, TX 77030, USA

## Abstract

Posttransplant lymphoproliferative disorder (PTLD) is a spectrum of diseases that involves abnormal lymphoid and/or plasmacytic proliferation in patients with solid organ or hematopoietic cell transplantation. It is a condition with a low incidence of 3.5–4.3% in liver transplant (LT) recipients. This case involves a 63-year-old male with history of LT for chronic HCV induced cirrhosis who presented with abdominal distension related to worsening ascites. Cytological ascitic fluid analysis revealed EBV (+) malignant cells without a malignant focal point on imaging. Diagnosis of monomorphic PTLD with primary effusion lymphoma-like morphology and immunophenotype was established. This case highlights the complexity in diagnosis, different diagnostic modalities, and rare clinical presentations of PTLD.

## 1. Introduction

Posttransplant lymphoproliferative disorder (PTLD) is a spectrum of diseases that involves abnormal lymphoid and/or plasmacytic proliferation in patients with solid organ or hematopoietic cell transplantation. It is a well-recognized condition with a relatively low incidence of 3.5–4.3% in liver transplant (LT) recipients [1, 2]. Degree of immunosuppression and EBV serostatus are the most common risk factors contributing to the development of PTLD. Diagnosis is made using tissue biopsy and in rare instances using body-fluid analysis. This case highlights the complexity in diagnosis, different diagnostic modalities, and rare clinical presentations of PTLD.

## 2. Case

**Figure 1:**
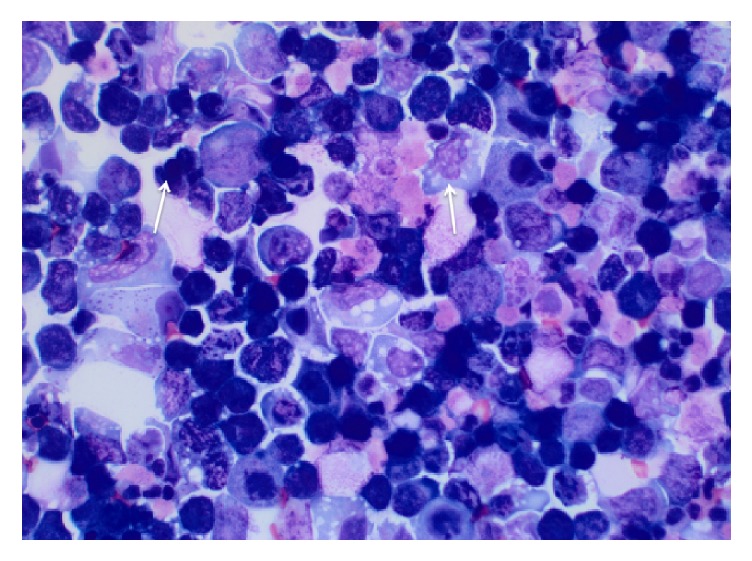
Ascitic fluid cytology with Diff-Quick stain demonstrating large atypical lymphocytes with round to anaplastic nuclei, dispersed chromatin, and basophilic cytoplasm (marked by the arrows). A subset of cells show plasmacytoid/plasmablastic morphology.

**Figure 2:**
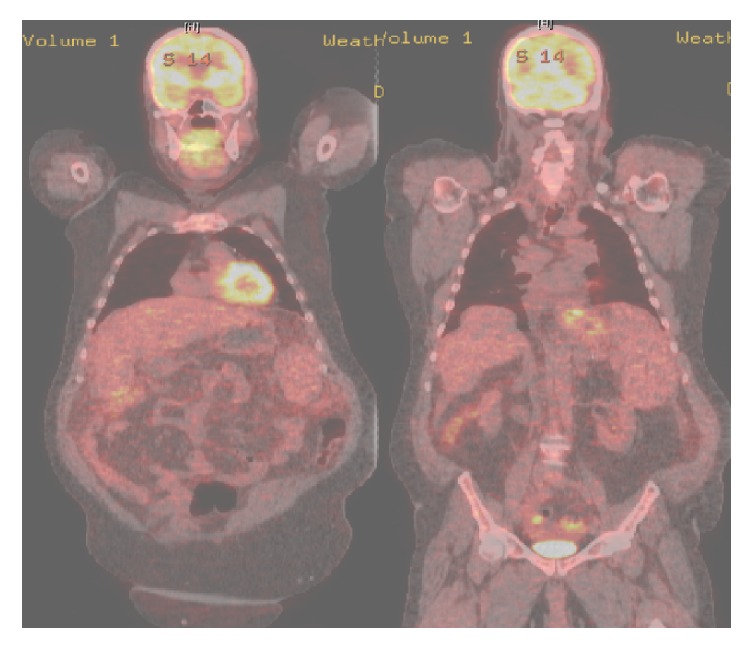
PET CT scan showing the absence of focal FDG uptake, in addition to stigmata of liver cirrhosis.

A 63-year-old Caucasian male with history of a LT for chronic hepatitis C (HCV) induced cirrhosis in 2004 presented with worsening abdominal distension. Patient developed HCV graft infection with resulting cirrhosis. In 2014, he was treated with Sofosbuvir and Simeprevir achieving sustained virologic response. His graft cirrhosis was complicated by mild ascites controlled with diuretics. On presentation, the patient was hemodynamically stable with a nontender and distended abdomen with fluid wave on exam. Laboratory testing on admission showed an elevated creatinine of 1.8 mg/dL (baseline 1.3 mg/dL), AST 39, ALT 36, ALP 113, and Total Bilirubin 2.3. Diagnostic paracentesis revealed serum-ascites albumin gradient > 1.1, protein level of 4.3 g/dl, RBC 4000 mm3, and WBC 2240 mm3 (1% PMN/61% Lymphocytes). Fluid cytology showed atypical pleomorphic malignant cells, a subset of which with plasmacytoid/plasmablastic morphology along with rare Hodgkin/Reed-Sternberg like morphology (Figure 1). Flow cytometry and immunohistochemical stain results demonstrated a T-cell predominant sample without aberrant markers for either T-cells or B-cells. Majority of cells were positive for MUM-1, EMA, and Ki-67 and negative for CD2, CD3, CD4, CD8, CD20, CD138, EBV-LMP1 (Epstein-Barr virus), HHV8, and so forth (Table 1). Clonal IgH gene rearrangement was negative. Given concern for a nonhematopoietic tumor and metastatic disease, a CT abdomen-pelvis and whole-body PET CT scan were obtained and a localized malignant focal point or lymphadenopathy was excluded (Figure 2). Tumor markers, including AFP, CEA, and CA 19-9, were within normal limits. HIV serology was negative.

During the admission, the patient's acute kidney injury improved after discontinuation of his diuretics and with volume resuscitation; however, his liver function worsened. The patient's maintenance immunosuppressive agent (Tacrolimus  .5 mg PO q12h) was increased to 1 mg PO q12h prior to discharge. With a growing concern for primary effusion lymphoma (PEL) in the setting of worsening ascites, cytogenetic analysis of the ascitic fluid was performed in this case given nondiagnostic cytology, immunostaining, and flow cytometry. An abnormal male karyotype with two clones with a t(8;14) translocation, along with multiple structural and numerical abnormalities, was noted. Epstein-Barr encoded region in situ hybridization on the ascitic fluid was positive within tumor cells. After secondary review at an outside institution, the patient was diagnosed with monomorphic PTLD with primary effusion lymphoma-like morphology and immunophenotype. His immunosuppressive therapy was discontinued during a posthospitalization clinic visit and he was referred to oncology.

Patient had a repeat staging PET scan with no FDG avid lymphadenopathy or visceral disease two months after the initial scan. In the setting of CD20 (−) disease, abnormal liver function tests, and poor performance status, the patient was started on Mini-CHOP at 50% dose reduction for 2 cycles every 21 days. His performance status continued to deteriorate and he required frequent therapeutic paracentesis despite chemotherapy. He underwent further dose reduction to 25% for 2 additional cycles. Patient was subsequently lost to follow-up one month after the last chemotherapy infusion.

## 3. Discussion

In patients with solid organ transplantation, PTLD is a common complicating malignancy. PTLD is classified into Benign Polyclonal Lymphoproliferation, Polymorphic PTLD, Monomorphic PTLD, and Classical Hodgkin Lymphoma-like PTLD, based on morphologic, immunophenotypic, genetic, and clinical features. Cellular proliferation observed in PTLD has been linked to the degree of chronic immunosuppression and decreased cell-mediated immunity. EBV and its encoded-factors, such as LMP1, have been associated with B-cell proliferation in cases of PTLD [3, 4]. Other associated risk factors include specific immunosuppressants (i.e., tacrolimus), age at the time of transplantation, and type of solid organ transplantation [5–7]. The 5-year incidence of PTLD in LT recipients is approximately 1-2%. Based on a single-center cohort study, the estimated incidence in LT patients was 4.3% over 17 years [1]. Another similar study found a disease prevalence of 3.5% [2].

Generally nodal or extranodal tissue biopsy following radiographic evaluation is the primary diagnostic methodology for PTLD. This process is complicated by the number of different clinical presentations of PTLD in LT patients. There have been rare instances where diagnosis via body-fluid analysis have been described in literature. A study evaluating cytology in posttransplant patients with histologically confirmed cases of PTLD, revealing 2/7 cases in which cellular proliferation and differentiation were revealed in ascitic fluid [8]. Another cohort study found 17 cases of post-LT PTLD with extranodal presentations involving the skin, gastrointestinal tract, urinary tract, and so forth. A malignant focal point was absent in 4 of these cases, with diagnostic fluid flow cytometry used in 2 subjects with ascites as their primary clinical manifestation [2]. There have also been a few case reports of PTLD occurring in pleural fluid [9].

Given the cytological findings and absence of a malignant focal point in this case, consideration was given to primary effusion lymphoma as the etiology of the worsening abdominal distension and ascites. Primary effusion lymphoma is a rare AIDs related non-Hodgkin's lymphoma with the pathophysiology linked to HHV8 and in many cases EBV. It mainly affects serosal surfaces including the peritoneum in 60–80% of cases [10]. Cytogenetic analysis of the ascitic fluid performed in this case reveled a translocation of chromosome 8 and chromosome 14 (c-myc) commonly seen in Burkitt's lymphoma. There have been cases of lymphomatous effusions, including extranodal Burkitt's lymphoma, in AIDs patients that were EBV (+) and HHV8 (−) that primarily involved serosal cavities [11]. This patient is both HIV and HHV8 negative, which makes PEL unlikely. Lymphomatous ascites has been described in post-LT patients; however, the cases of Burkitt's lymphoma involved intra-abdominal/pelvic masses [2]. There have also been rare cases of PTLD that mimic primary effusion lymphoma in EBV (−) and HHV8 (−) patients [12]. In a gene analysis profile study, the impact of HHV8 and EBV on cellular gene expression and pathogenesis of lymphomatous effusions was evaluated. Based on their findings, EBV (−)/HHV8 (−) patients have a distinct set of gene expression that differs from EBV (+)/HHV8 (−) patients. The latter group were found to induce translocation involving the c-myc locus [13]. This patient was diagnosed with monomorphic PTLD with primary effusion lymphoma-like morphology and immunophenotype.

Lymphoproliferative disorders in posttransplant patients are a well-recognized phenomenon. However, without clear indication of nodal or extranodal involvement, it is important to recognize the possibility of malignant proliferation within body cavities/body fluids and the other clinical presentations. Therapy initially involves deescalation of immunosuppression, followed by the initiation of immunotherapy (i.e., Rituximab for CD20 (+) disease), chemotherapy, and/or radiation. This case highlights the complexity in diagnosis, different diagnostic modalities, and rare clinical presentations of PTLD.

## Figures and Tables

**Table 1:** Ascitic fluid immunohistochemical staining results.

Positive	MUM-1, EMA, and Ki-67

Negative	CD45, CD2, CD3, CD4, CD5, CD7, CD8, CD15, CD20, CD30, CD43, CD56, CD138, PAX5, TDT, HHV8, ALK1, myeloperoxidase, pan keratin, CAM5.2, CK5/6, CK7, CK20, monoclonal CEA, hepatocyte, glypican 3, D2-40, calretinin, S100, SMA, desmin, myogenin, EBV-LMP1, and HHV8

## Abbreviations

PTLD: Posttransplant lymphoproliferative disorder
LT: Liver transplant
HCV: Hepatitis C
EBV: Epstein-Barr virus
HHV: Human herpesvirus.
